# Unveiling kratom's dark side: Case report implicating the botanical ingredient as a culprit in recalcitrant photo-exposed hyperpigmentation

**DOI:** 10.1016/j.jdcr.2025.02.017

**Published:** 2025-03-10

**Authors:** Kostandin Valle, Kaleigh Wingate, Daniel Tinker, Duane Dilworth

**Affiliations:** a University of Missouri School of Medicine, Columbia, Missouri; b Florida State University College of Medicine, Tallahassee, Florida; c Deluxe Dermatology LLC, Saint Louis, Missouri

**Keywords:** chemical peel, hyperpigmentation, kratom, melanin, Mitragyna speciosa, photodistributed rash, substance-induced hyperpigmentation

## Introduction

Kratom (*Mitragyna speciosa*) is a plant native to Southeast Asia with opioid-like properties at high doses and stimulant-like properties at low doses. One of kratom's major uses is as a cost-effective method of self-treatment for opioid withdrawal. However, just like opioids and stimulants, it has addictive properties and can precipitate dependence or withdrawal after continuous use.^1^ The main alkaloids studied within kratom are mitragynine and 7-hydroxymitragynine, with the former comprising over 60% of the total alkaloids within the plant leaf. Upon human consumption, mitragynine is metabolized into 7-hydroxymitragynine, the more biochemically active form, within the cytochrome P450 system in the liver.^2^ These compounds exert various effects on the body by acting on numerous receptors including mu-opioid, serotonin, and dopamine, and inhibiting parasympathetic effects.^1^^,^^3^ Unlike opioids or stimulants, kratom does not appear on regular urine drug screens. As such, it has the potential to become a desired alternative product.^3^

Detailed history-taking is imperative in detecting kratom use in patient populations. In 2020, over 2 million people in the United States endorsed the use of kratom for its psychogenic effects. Now, the substance can be bought in various forms with increasing accessibility as companies, such as CBD Kratom, have increasingly begun marketing kratom-infused products.^1^^,^^3^ To date, there are few studies in the United States on the full effects of kratom. However, some sources estimate that 20% of users experience kratom use disorder and a multitude of adverse effects, including psychosis, shortness of breath, hypertension, hepatic injury, hypothyroidism, and various other systemic symptoms.^3^ We highlight an underrecognized dermatologic manifestation of kratom, photo-distributed hyperpigmentation, for which few written reports showcase a similar manner of presentation (Table I).^2^^,^^4^, ^5^, ^6^

**Table I:** Summary of existing case reports highlighting kratom-induced hyperpigmentation

Patient	History and clinical course	Histopathology findings	Treatment	Source
54-y-old Caucasian male	Four-to-five-year history of kratom ingestion. Presented with hyperpigmentation on the forearms, hands, ears, and cheeks.	Epidermal red-brown intracellular and interstitial pigment deposition in regions with solar elastosis. Fontana-Masson stain was positive.	N/A	Powell L.R. et al
56-y-old female	Seven-year history of kratom consumption for chronic pain. Presented with blue-gray patches of hyperpigmentation on the extremities, face, chest, and neck.	Red-brown pigment deposition within dermal histiocytes. Fontana-Masson stain was negative.	Kratom cessation and compounded 12% hydroquinone, 6% Kojic acid, 2% niacinamide, and 1% ascorbic acid. Treatment failed.	Johnson K.M. et al
62-y-old male	One-year history of diffuse tender, pruritic, hyperpigmented skin lesions in sun-exposed areas, including the face, neck, and forearms.	Superficial perivascular lymphocytic infiltrate and numerous melanin-containing macrophages in the superficial dermis. Fontana-Masson stain was positive, and iron stain was negative.	Kratom cessation and sun protection. Patient was lost to follow-up.	Gandhi I. et al
30-y-old male	Five-year history of kratom use, 3-7 grams per day. Presented with dark gray-blue discoloration on the cheeks, back of the neck, dorsal hands and forearms, vision changes, and anxiety.	N/A	Kratom cessation 16 mo prior to visit. Suggestion of Q switch lasers for treatment based on other hyperpigmentation disorders.	Patel S. and Phelan N.

Hyperpigmentation as a side effect of substance or drug use is not uncommon, and some of the most common culprits include minocycline, amiodarone, antipsychotics like phenothiazines, and antineoplastic drugs.^7^ The mechanism by which these drugs cause hyperpigmentation varies but includes drug accumulation within melanocytes, oxidative stress with resultant lipofuscin deposition, stimulation of local melanin synthesis, or disruption of melanosome formation and melanin transport. While the exact mechanism by which kratom causes hyperpigmentation is unknown, various hypotheses exist that differ from the mechanisms currently known in other drug-induced forms of hyperpigmentation. One hypothesis is that mitragynine and 7-hydroxymitragynine stimulate melanocyte activity via mu-opioid receptor agonism, like beta-endorphin activity seen endogenously.^8^ Another hypothesis is that the antagonistic effects of mitragynine on D2 receptors within the pituitary gland may stimulate the production of alpha-melanocyte-stimulating hormone and other proopiomelanocortin-derived peptides, thereby contributing to hyperpigmentation.^9^

## Our report

**Fig 1:**
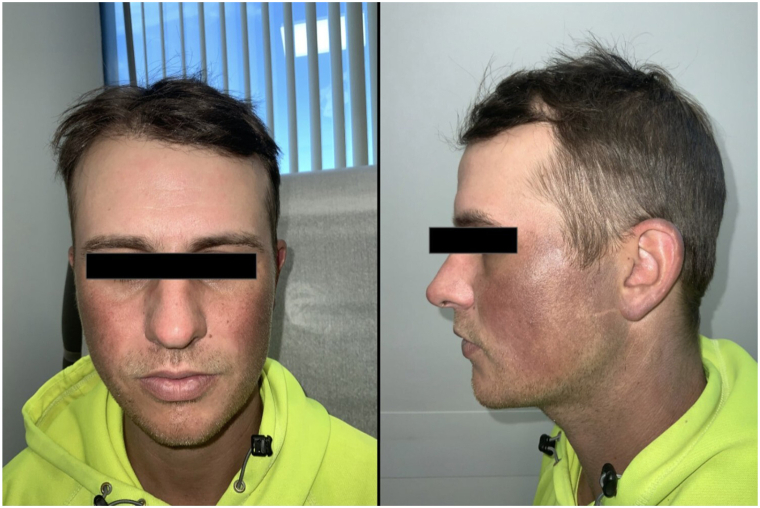
Diffuse, symmetric, deeply grey-to-violaceous hyperpigmented pathces in a photo-exposed distribution on the face.

We present a 32-year-old male with a 1-year history of recalcitrant gray-to-blue-brown patches with underlying telangiectasias on the face, ears, and lateral and posterior neck. (Fig 1). When the patient first presented to the clinic, erythematotelangiectatic rosacea and melasma were suspected. A broad-spectrum sunscreen was recommended, as well as oxymetazoline 1%/ivermectin 1%/niacinamide 2% cream and hydroquinone 12%/kojic acid 6%/vitamin C 1%/niacinamide 2% cream.

Three months after his initial visit, the patient reported that hyperpigmentation on his cheeks had improved slightly, but the rest of the affected areas remained the same. The patient was advised to discontinue hydroquinone and to continue using the oxymetazoline cream. Due to the persistence of his hyperpigmentation, melasma was favored as the primary diagnosis, and the patient was prescribed tranexamic acid 325 mg twice a day.

After 4 months, the patient returned without any improvement in his hyperpigmentation. He endorsed the continued use of sunscreen and wearing a hat for sun protection, with a notable lack of involvement in areas by the hat. Further history was obtained, and the patient revealed that he had been employed by a water company for 1 year before the onset of hyperpigmentation. The patient's duties included water delivery, digging soil, reading meters, and working outside. Due to the patient's occupational exposure, Riehl's melanosis was considered a top differential, and a punch biopsy was performed.

**Fig 2:**
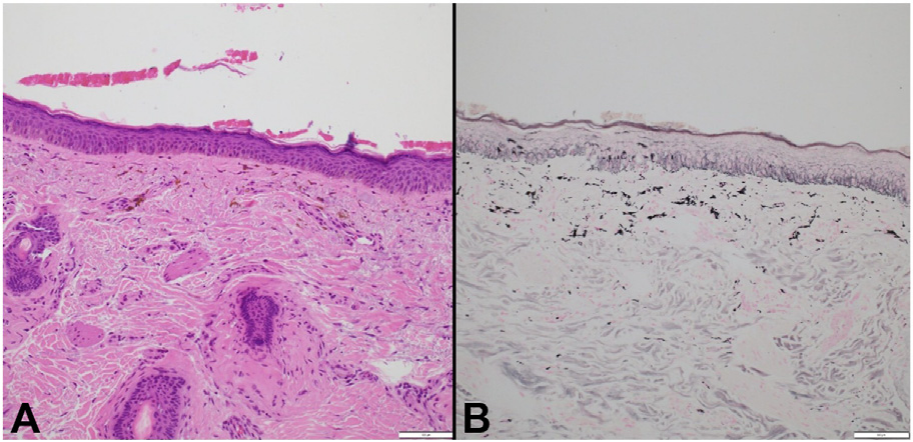
**A,** Hematoxylin and eosin stain showing deposition of brown pigment in the superficial papillary dermis and within epithelioid histiocytes. **B,** Fontana-Masson stain demonstrating melanin pigment within histiocytes and the interstitium of the superficial papillary dermis.

Hematoxylin and eosin stain revealed brown pigment in the superficial papillary dermis and within epithelioid histiocytes (Fig 2, *A*). Fontana-Masson stain was positive, confirming the presence of melanin pigment. (Fig 2, *B*). Postinflammatory pigment changes were evident. Upon review of the patient's supplements, it was revealed that kratom was being consumed. The patient had begun using kratom 3 times daily for relaxation before developing his symptoms; however, he did not detail the dosage or route of administration.

## Discussion

Plant-induced dermatological effects are well-documented; however, kratom-induced hyperpigmentation is an underrecognized and underreported entity. Early recognition is essential to guide proper management. We underscore the importance of thorough history-taking and increasing vigilance in recognizing kratom's ability to induce recalcitrant hyperpigmentation. All reported cases are in photo-exposed anatomic locations, but further research is needed to elucidate the mechanism.

Nevertheless, dermatologists must be aware of this increasingly available and popular plant extract. We underscore a heightened index of suspicion in patients who present with hyperpigmentation that fits the clinical picture outlined in this summary. Stopping use is imperative for reversing hyperpigmentation. However, we must be sensitive to its addictive nature and the discomfort of withdrawal. Abstention may be difficult without professional support and a comprehensive treatment plan.

Chemical peels are a widely used therapy for hyperpigmentation due to their ability to remove various layers of the skin. One commonly used medium-depth peel for hyperpigmentation includes Jessner's peel, which consists of salicylic acid, lactic acid, and resorcinol in an alcohol solution, and has been shown to decrease hyperpigmentation in patients with melasma significantly.^10^ Additionally, trichloroacetic acid peels have also been shown to be effective in the treatment of hyperpigmentation due to their medium-depth peel ability.^10^ Given the nature of our patient's hyperpigmentation, as well as other reported cases, our management consists of medium-depth chemical peels containing salicylic acid, lactic acid, trichloroacetic acid, and croton oil preconditioned with 8% hydroquinone. This peel was specifically compounded in-office, but the active ingredient concentrations parallel that of other medium-depth peels. The patient has yet to follow-up for evaluation.

## Conflicts of interest

Dr Dilworth is a speaker for Galderma, Lilly, and Pfizer; is a consultant for Sanofi; and is an advisor for Bristol Myers Squibb. Author Valle, Drs Wingate, and Tinker have no conflicts of interest to declare.
